# The Foreign Journals: Midwifery

**Published:** 1840-01

**Authors:** 


### MIDWIFERY.

### *Case of Metritis with Epilepsy, in which Separation and Expulsion of a great part of the Vagina and of the Neck of the Uterus, followed by perfect Recovery, took place.* By Dr. ANTONIO LONGHI.

ROSA GATTI, æt. 27, was admitted, May 11, 1835, to the Milan Hospital. Her health had, with the exception of occasional epileptiform attacks, hitherto been excellent. The catamenia had appeared early, and were extremely copious, appearing sometimes twice a month. In February, 1835, suppression took place, without causing any serious inconvenience to the patient, and still existed at the time of her admission. Immoderately addicted to drinking and sexual indulgence, her propensity for the latter had of late been additionally excited by pruritus of the genitals. On the 8th of May she was seized with an epileptic fit of much greater intensity than usual; for this she had been twice bled, previous to her admission. When examined at the hospital, there was total loss of sensation and motion, with a comotose state; uterus somewhat enlarged, and found per vaginam, to be of the size usual at the third month of pregnancy; fetid yellow discharge from vagina; violent convulsions brought on by abdominal pressure. Copious bleeding was immediately ordered, and repeated on the 12th and 13th; the frequency of the epileptic paroxysms was thereby decreased, but all the other symptoms remained unchanged. After the application of eighteen leeches to the head, of four blisters, cupping at the occiput, &c., the coma had, on the 17th, in a great measure disappeared, and the patient answered some questions. The abdominal tenderness, however, increasing on the 18th, twenty-four leeches were applied to its most painful part, the epigastrium, and a grain of tartar emetic given in an emulsion, which was immediately rejected by vomiting. There still remained an increasing febrile movement; the pulse was hard and small; and lancinating pains were felt in the abdomen. On the 21st, in straining, as if to discharge the contents of the rectum, she felt a voluminous body pass through the vagina, which, as she fancied herself pregnant, she took for a fœtus. This body, on examination, was found of pyriform shape, with a semicircular aperture at the apex, in size somewhat larger than the healthy unimpregnated uterus, fetid, blackish, of moderate consistence at the base, from which part its cohesion diminished towards the apex. Carefully washed, it appeared of a dirty white colour, and covered with soft cellular membrane, the base rounded and smooth, with a small central opening. On introducing the finger within the great aperture at the apex, a large cavity was felt, terminating at the opposite end by a fleshy body; and on slitting up the walls of this cavity, they were found to consist of a part of the vaginal parietes, and the fleshy body of the neck of the uterus. The patient, still believing she had miscarried, obstinately refused all medicine. Emollient vaginal injections, however, were constantly employed, and a milk diet ordered. Under this simple treatment the discharge from the vagina gradually decreased, as did also the fever and abdominal symptoms; and on the 18th of June the patient was discharged in perfect health, with the exception of a slight yellow inodorous discharge. On this day, a careful examination, per vaginam, was for the first time made. The external parts were natural, the vagina notably contracted, especially at the upper part. At about the ordinary height it ended in a firm cartilaginiform ring, that scarcely allowed the passage of the index finger. Beyond this ring the finger entered a cavity, of considerable width, supposed to have been formed by the separation of the parts passed by the vagina. The finger, pushed still higher, touched two unequal small prominences, divided by a transverse fissure. The entire cavity was surrounded by a wall of lardaceous consistence, without callosity or ulceration. Immediately on leaving the hospital the patient returned to her old habits; but the pain and hemorrhage attending copulation were so great, the first day, that she was obliged to desist. The vagina, however, gradually yielding, ceased to interfere with the gratification of her desires. Towards the end of June the menses reappeared, and flowed with the utmost regularity. The parts discharged are preserved at the Milan hospital.

*Giornale delle Scienze Med.-Chir.* No. xxii.

---

### *Case of Impacted Head, with great subsequent Loss of Parts.*
By M. Cazenave, of Bordeaux.

The patient, a primipara, æt. twenty-five, of diminutive stature, had always enjoyed good health. As the labour did not advance, she was bled, had a bath, and took some ergot of rye; but in spite of pains, which continued for thirty-six hours, no change was produced. After repeated attempts to apply the forceps, a second practitioner was called in, and he not succeeding better than the first, a third, and afterwards a fourth, were summoned to her assistance. They having tried in vain to apply the forceps, it was determined to turn the child, and this was effected with great difficulty, from the violence of the pains, and liquor amnii having escaped thirty-six hours previously. The body and arms were extracted, but the head remained impacted, in spite of every effort to disengage it. A fifth practitioner was now added, but he was not more fortunate in his attempts. It being now midnight, they determined to let her rest till the morning, hoping that fresh contractions would flatten the head and expel it. Accordingly, the next morning, the head was found in the vagina, and easily extracted. The labia swelled, and the woman was attacked with metro-peritonitis and diarrhœa; general sloughing followed, large portions of ragged slough came away, and continued to do so until the 20th day. On seeing her nearly a month afterwards, M. Cazenave could find no traces of the labia, nymphæ, vagina, perineum, or anus; one large opening extended from the meatus urinarius to the coccyx, and showed that the recto-vaginal septum and lower part of the rectum were all destroyed; the urine did not pass by the urethra, and the fæces continually accumulated in the cloaca which had thus been formed. Further examinations showed, also, that about two inches and a half of the rectum had been destroyed, and that the neck of the bladder was obliterated. Under the use of topical bathing, injections, and mild nourishing diet, the health improved considerably, and the parietes of the huge cavity contracted somewhat; she was also enabled to pass a little urine by the urethra; still, however, the constant passage of fæces and urine by the same opening produced severe inflammation of the part, with all the sympathetic derangements attendant upon such irritation, the health began to fail, and in this state of suffering she has remained up to the present time.

[We need hardly say that perforation was most strongly indicated here, and would have doubtless spared her such dreadful suffering and injury: what object could possibly be gained by avoiding this operation? It could scarcely be any rational expectation of saving the child's life after so long and violent a labour.] —M. Cazenave has extracted a very curious and somewhat similar case from the American Journal of Medical Science for August: "A stout woman, ætat. thirty, was delivered of her first child after a labour of thirty-six hours' duration, during which she was bled, narcotized with opium, and lacerated with a pair of bad forceps: in four days after, the labia were found in a gangrenous state, for which cinchona and charcoal were ordered; this was followed by tympanites, which yielded to castor-oil, ablution with tepid water, &c. On the 19th day the fundus uteri descended into the vagina, and presented itself at the os externum, and with a little assistance on the part of the nurse the whole uterine apparatus was removed. The rectum separated at some inches above the pubes, and its lower portion depended between the labia; this the nurse also drew down in the presence of the practitioner, and it separated at the sphincter ani. On examining the parts, he found a single opening, extending from the coccyx to the pubes. Some time afterwards, the end of the intestine descended several inches, and hung loosely on the concave surface of the sacrum; a sponge was introduced into the cavity to support the rectum and prevent the access of air. The destruction of the parts was so complete, and the opening so large, as to bring within view the whole inner surface of the pelvis; in spite of which, after protracted suppuration, the wound cicatrized from behind forwards; her health gradually returned, and she would now feel quite well, but for the inability to retain her urine and fæces. In six months after labour the secretion of milk established itself, and lasted two months, without influencing the other functions."

*L'Expérience.* No. lxxxiv. *Fevrier* 7, 1839.

---

### *Case of Occlusion of the Neck of the Uterus during Parturition.*
By FELIX HATIN, M.D.

MADAME D., ætatis thirty-six, pregnant of her first child, felt labour-pains during the day of July 12, 1837. I was sent for at ten o'clock, p.m., and found the water had escaped, not in a single gush, but in small quantities, with successive pains; the uterine contractions were still slight, and occurring at distant intervals. I found the cervix flaccid, but much more prominent than usual, and could not discover any trace of orifice. At five, a.m., the cervix was effaced, but still no opening was to be found. I felt distinctly a round, smooth, voluminous mass plunging into the cavity of the pelvis, but nothing more. As no bad symptom appeared, I determined to wait until the tumour referred to should come down lower; it did so, and proved to be the fœtal head invested by the uterus. I felt convinced of the absence of the uterine orifice; but before practising the vaginal Cæsarian operation, I made the most minute search for the fissure through which conception and the escape of the liquor amnii had been effected. I felt a small spot, a little less resisting than the rest of the surface, and passed a female catheter up to it along the finger. By pressing gently, I carried this through the thin part, and then, substituting my finger for the instrument, widened the breach; the sensation produced was precisely that of a thin membrane tearing. The neck then dilated, the head presented in the first position, and, half an hour after, the labour terminated naturally.

[There is a deficiency of detail in this narrative; but the case furnishes a good example of the success of the treatment particularly recommended by Nägele.] *L'Expérience. Mai*, 1839.

